# Dynamic monitor of CT scan within short interval in invasive pulmonary aspergillosis for nonneutropenic patients: a retrospective analysis in two centers

**DOI:** 10.1186/s12890-021-01512-8

**Published:** 2021-04-30

**Authors:** Fei Chen, Yonghong Zhong, Na Li, Huijie Wang, Yanbin Tan, Hao Zhang, Wen Hua, Yanxiong Mao, Huaqiong Huang

**Affiliations:** 1 Key Laboratory of Respiratory Disease of Zhejiang Province, Department of Respiratory and Critical Care Medicine, Second Affiliated Hospital of Zhejiang University School of Medicine, Hangzhou, Zhejiang China; 2 Yuhang Branch, the Second Affiliated Hospital, Zhejiang University School of medicine, Zhejiang Hangzhou, China; 3 Department of Radiology, Second Affiliated Hospital of Zhejiang University School of Medicine, Hangzhou, Zhejiang China

**Keywords:** CT dynamic monitoring, Aggressive pulmonary aspergillosis, Nonneutropenic, Chronic respiratory diseases

## Abstract

**Background:**

In nonneutropenic patients with underlying respiratory diseases (URD), invasive pulmonary aspergillosis (IPA) is a life-threatening disease. Yet establishing early diagnosis in those patients remains quite a challenge.

**Methods:**

A retrospective series of nonneutropenic patients with probable or proven IPA were reviewed from January 2014 to May 2018 in Department of Respiratory Medicine of two Chinese hospitals. Those patients were suspected of IPA and underwent lung computed tomography (CT) scans twice within 5–21 days. The items required for IPA diagnosis were assessed by their host factors, mycological findings and CT scans according to the European Organization for Research and Treatment of Cancer (EORTC) and the National Institute of Allergy and Infectious Diseases Mycoses Study Group (MSG) criteria (EORTC/MSG criteria).

**Results:**

Together with the risk factors, mycological findings and nonspecific radiological signs on first CT, ten patients were suspected of IPA. With the appearance of cavities on second CT scan in the following days, all patients met the criteria of probable or possible IPA. Except one patient who refused antifungal treatment, nine patients received timely antifungal treatment and recovered well. One of the nine treated IPA cases was further confirmed by pathology, one was confirmed by biopsy.

**Conclusions:**

Dynamic monitor of CT scan provided specific image evidences for IPA diagnosis. This novel finding might provide a noninvasive and efficient strategy in IPA diagnosis with URD.

## Introduction

Invasive pulmonary aspergillosis (IPA) is a fungal infection which is the most common form of invasive aspergillosis and a cause of mortality. IPA usually affects immunocompromised individuals such as solid organ transplant recipients and patients with hematological malignancies including hematopoietic stem cell transplant recipients. Some research results show that IPA is associated with significant morbidity and carries a crude mortality rate of up to 30–40% in some risk groups [1, 2]. Aside from those high-risk groups, the incidence of IPA in nonneutropenic patients with underlying respiratory diseases (URD) such as chronic obstructive pulmonary disease (COPD), asthma, lung cancer or autoimmune diseases with pulmonary involvement is increasing [3–5]. Patients with COPD were reported to be most vulnerable for IPA development [4, 6]. The mortality of IPA in URD patients has been found to be between 32% and 100% [6–8]. Patients with URD have similar symptoms, signs and radiology, which is likely to cause missed or misdiagnosed IPA in clinical diagnosis [2–4].

Due to the lack of specificity of the clinical manifestations of IPA, early diagnosis is difficult and the treatment effect is poor, so the mortality rate is extremely high. To establish diagnosis of IPA in URD patients without the classic risk factors is usually difficult, although several diagnostic criteria such as EORTC/ MSG Criteria [9] and Bulpa Criteria had been applied in daily practice [10]. IPA patients with URD usually present severe clinical conditions and poor lung function which make it difficult to obtain sterile lower respiratory tract (LRT) samples by bronchoscopy. So sterile samples are rarely collected in daily practice, despite they are important for IPA diagnosis. Non-specific symptoms and signs and insufficient accuracy of diagnostic tests delay early identification and timely antifungal treatment, which leading to an increased physical and psychological burden.

Novel tests for diagnosis of IPA in patients with URD are under development. Next-generation sequencing (NGS), aspergillus-specific lateral-flow device tests, bioluminescence and small molecule imaging were reported to be helpful in diagnosis of IPA [7, 11, 12]. However, these novel tests need to be verified in large population and the cost of the tests is very high. A simple, noninvasive and effective diagnosis method is urgently needed, especially in developing countries.

Here by adopting a strategy of repeated CT scans within a short interval, we identified 10 cases of IPA in nonneutropenic patients. Our data showed that IPA had common imaging signs such as consolidation and tree-in-bud pattern in the early stage, then showed typical IPA signs such as cavitated nodules and halo signs in the following days. Dynamic CT review within a short interval provided more available evidence for EORTC/ MSG criteria. This strategy might be useful in diagnosis of IPA for non-hematologic immunocompromised patients.

## Materials and methods

We retrospectively assessed patients with invasive pulmonary aspergillosis admitted in Department of Respiratory Medicine of First People’s Hospital of Yuhang District and Second Affiliated Hospital of Zhejiang University School of Medicine respectively, between Jan 2014 and May 2018, and recruited the patients who had chest CT more than or equal to twice during the onset and whole hospitalization.

The EORTC/ MSG criteria [9] was taken as IPA diagnostic criteria (Table 1). We added URD history as host factor according to the previous study [13]. Patients were classified into proven, probable or possible IPA based on host factors, clinical data, mycological criteria, histopathological or cytopathological examination. Details as follows:

- Possible cases required host factors and clinical data but without *Aspergillus* isolation or serology. Written informed consent was obtained from each patient.
- Probable cases require host factors, clinical data (meet one of the followings in CT: dense and well-circumscribed lesion with or without a halo sign, an air-crescent sign or a cavity), and microbiological factors (isolation of *Aspergillus* in LRT samples, or positive serum or bronchoalveolar lavage fluid (BALF) Galactomannan test (GM tests).
- Proven IPA identification requires histopathological or cytopathological examination of lung tissue showing *Aspergillus* hyphae from needle aspiration or biopsy specimen with evidence of associated tissue damage, or positive culture for *Aspergillus* from a sample obtained by sterile procedure from the lung.

**Table 1:** IPA classification according to revised 2008 EORTC/MSG criteria

EORTC/MSG criteria	Host factors	Clinical criteria	Mycological criteria	Histopathological or cytopathological examination
	Recent history of neutropenia Receipt of an allogeneic stem cell transplant Prolonged use of corticosteroids Treatment with other recognized T cell immunosuppressants Inherited severe immunodeficiency URD*	The presence of 1 of the following 3 signs on CT: Dense, well-circumscribed lesion(s) with or without a halo sign An air-crescent sign A cavity	Positive culture and/or microscopy result for sputum, BALF, bronchial brush Positive serum or BALF GM tests	Histopathologic or direct microscopic demonstration
Possible IPA	√	√		
Probable IPA	√	√	√	
Proven IPA				√

*URD* underlying respiratory diseases, *LRTs* lower respiratory tracts, *BALF* bronchoalveolar lavage fluid, *GM* galactomannan tests

*URD was added as host factors in our study according to previous report

This study was approved by institutional review board of both hospitals. All procedures performed in studies involving human participants were in accordance with the Helsinki Declaration. Written informed consent was obtained from the patient.

## Results

Ten patients were diagnosed as uncertain IPA at first when they showed poor response to broad antibiotic and/or systemic corticosteroids (Table 2).

**Table 2:** Patients’ characteristics

Case no.	Sex		Age	Diagnosis while admission	Comorbidity	Smoking (package/year)	Methylprednisolone consumption before diagnosis (mg)	Broad antibiotic therapy	Neutrophil (×10^9^)	Sputum culture (times)	Sputum culture
1	M		71	Pneumonia	Coronary heart disease, Hypertension, DM,COPD	55	253	Yes	10.37	4	*Aspergillus* (once) *Klebsiella* (twice) Normal (twice)
2	M		77	Pneumonia	Hypertension, prostatic hyperplasia, prostatic cancer	N/A	1048	Yes	12.22	1	Normal (once)
3		M	73	Acute exacerbation of asthma	Hypertension, DM, rheumatoid arthritis	20	1040	Yes	13.44	7	Normal (seven times)
4		F	81	Acute exacerbation of COPD	Zoster, schistosomiasis liver disease	N/A	1032	Yes	9.96	2	*Stenotrophomonas maltophilia*(once) Normal (once)
5		F	62	Acute exacerbation of asthma	None	N/A	2360	Yes	5.47	5	*Filamentous fungi* (once) *Klebsiella *(once) *Acinetobacter Bauman* (once) Normal(thrice)
6		M	58	Pneumonia	Postoperative esophagus cancer	20	None	Yes	8.79	5	*Candida albicans* (twice) Normal(thrice)
7		M	84	Pneumonia	DM, knee arthroplasty	N/A	1200 mg	Yes	17.81	1	*Aspergillus*(once)
8		M	64	Pneumonia	Liver dysfunction	30	None	Yes	9.13	3	*Aspergillus* (twice) Normal(once)
9		M	76	Pneumonia	Hypertension\ Parkinson \COPD	15	None	Yes	2.52	1	Normal(once)
10		M	42	Pneumonia	Drug—induced hypersensitivity syndrome / Hypertension/DM	20	Unclear dosage for more than 2 months,	Yes	4.31	2	*Candida albicans* (twice) *Staphylococcus aureus* (once)

Table 2 is the basic information of the patients. Gender: 8 males and 2 females; Reasons for admission: 1 patient with acute exacerbation of COPD, 2 patients with acute exacerbation of asthma, 7 patients with pneumonia; previous medical history: 1 patient with a history of esophageal cancer, 1 patient with a history of prostate cancer, 1 patient with herpes zoster infection (relapse 2 months before admission); comorbidities: 1 patient with coronary heart disease, 5 patients with hypertension, and diabetes mellitus (DM) 4 cases, 2 patients with COPD. There were 6 patients with smoking history. The rest had no history of malignant tumors, hematological malignancy or long-term use of immunosuppressive agents. None of the patients had rheumatoid arthritis treated with corticosteroids or immunosuppressive agents, nor had prostate cancer and esophageal cancer after chemotherapy.

The physical examination results showed: all patients had cough and sputum, eight patients had wheezing symptoms, some of them had thick wet rales, 4 patients had dyspnea, and 5 patients had body temperature between 37.5 and 39.1 °C. All patients had poor response to broad-spectrum antibiotic and/or systemic corticosteroids. Two of them were admitted to the intensive care unit (ICU).

Sputum culture was ordered for once at least and six times at most before initiation of antifungal therapy. Sputum culture results showed: Three patients’ sputum cultures revealed *Aspergillus* once or twice. One patient’s sputum sample reported *filamentous fungi* in all six sputum samples. Six patients’ cultures did not reveal *Aspergillus* at all. In addition, four patients had a positive galactomannan (GM) test in blood.

In this study, only one patient underwent bronchoscopy, six patients were considered intolerant to bronchoscopy by their attending doctors, and two refused bronchoscopy. Another patient received CT-guided lung biopsy first, which confirmed the diagnosis of IPA. As a result, bronchoscopy was unnecessary for him.

**Fig. 1:**
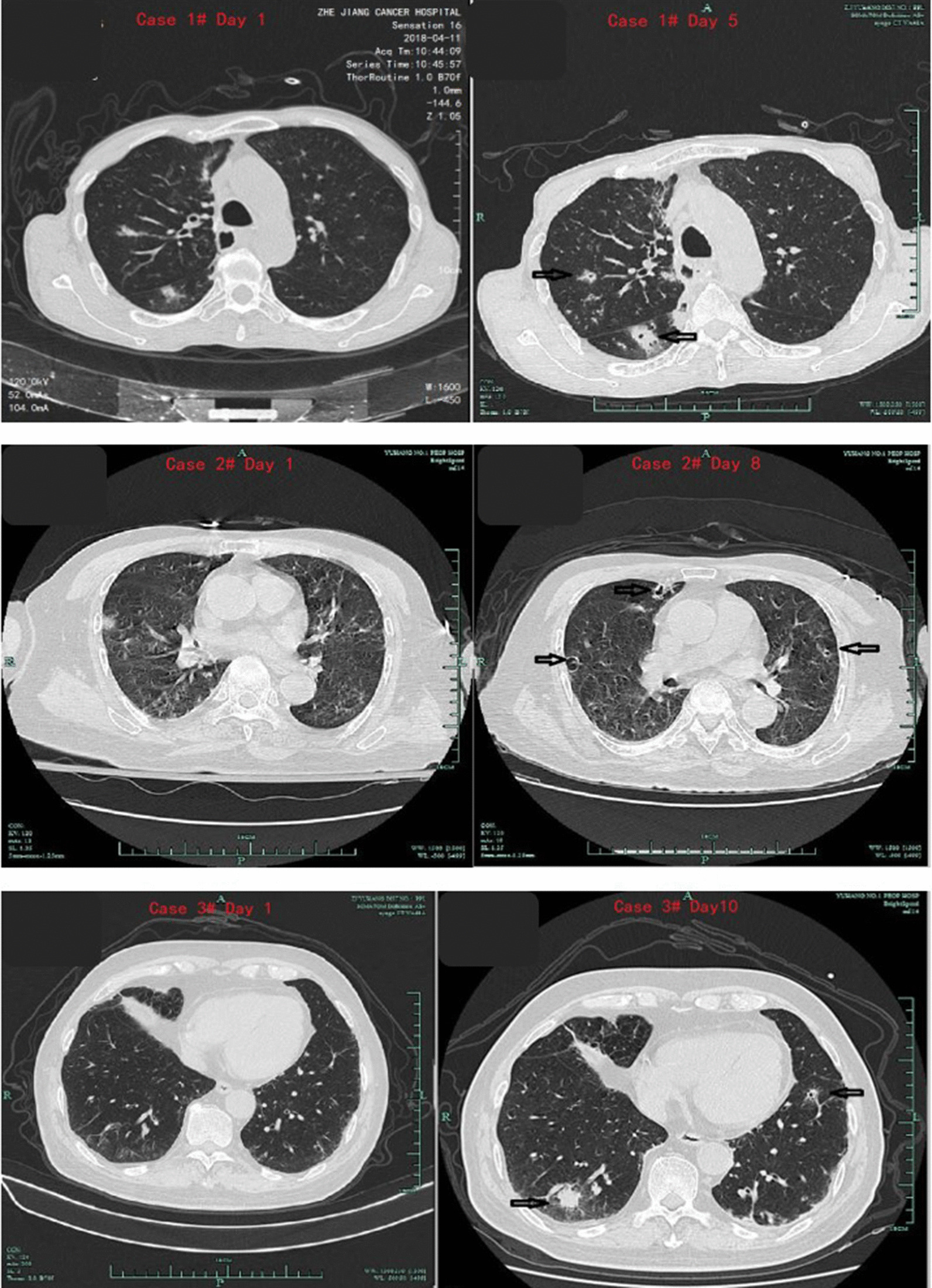
Dynamic monitoring of CT scans at short intervals of 5, 8 and 10 days in three cases respectively. The first CT scan of patients showed nonspecific signs. The second CT scan had deterioration with multiple nodules and cavities. The black arrows showed the specific signs of nodular and cavity

CT scan results showed: all ten patients underwent chest CT scan twice. A radiologist was invited to review the CT signs in a single-blind way (Tables 3, 4). At the first CT scan, patients showed common signs of inflammation, like scattered peri-bronchial consolidations, thickening of bilateral lung texture, small nodular lesions along the bronchial tree, and the ‘tree-in bud’ pattern. Second lung CT scan was ordered at short intervals of 5–22 days (averaging 9.7 days). All patients had deterioration of lesions with several nodules and cavities. The changes of lung CT in 3 patients are shown in Fig. 1. The intervals of CT scan were 5, 8 and 10 days in three cases respectively.

**Table 3:** The signs appearing in the initial CT scans

Case no.	Peri-bronchial consolidations	Thickening of bilateral lung texture	The ‘tree-in bud’ pattern	Big nodular infiltrates (≥ 3 cm)	Tiny-small nodular infiltrates (< 3 cm)	Halo sign	Cavities
1	√	√	√	×	√	√*	×
2	√	√	√	×	√	×	×
3	√	√	√	×	×	×	×
4	√	√	√	×	×	×	×
5	√	√	√	×	×	×	×
6	√	√	√	×	√	×	×
7	√	√	√	×	×	×	×
8	√	√	√	×	√	√	×
9	×	√	×	√	√	×	×
10	√	√	√	×	√	×	×
Total number	9	10	9	1	6	2	0

A radiologist was invited to review the patients’ CT scans in a single-blind way. “√”, the sign was found on CT. “×”, didn’t found on CT

*There was a small nodule (1.1 mm × 0.9 mm) with halo sign in upper right lung on CT scan

**Table 4:** The signs appearing in the second CT scans

Case no.	Peri-bronchial consolidations	Thickening of bilateral lung texture	The ‘tree-in bud’ pattern	Big nodular infiltrates (≥ 3 cm)	Tiny-small nodular infiltrates (< 3 cm)	Halo sign	Cavities	The intervals between initial and second CT scan (days)
1	√	√	√	×	√	√*	√	6
2	√	√	√	×	√	×	√	8
3	√	√	√	×	×	×	√	10
4	√	√	√	×	×	×	√	7
5	√	√	√	×	×	×	√	20
6	√	√	√	×	√	×	√	5
7	√	√	√	×	×	×	√	5
8	√	√	√	×	√	√	√	7
9	×	√	×	√	√	×	√	22
10	√	√	√	×	√	×	√	7
Total number	9	10	9	1	6	2	10	9.7^#^

A radiologist was invited to review the patients’ CT scans in a single-blind way. “√”, the sign was found on CT. “×”, didn’t found on CT

^#^The averaging intervals between initial and second CT scan (days)

**Fig. 2:**
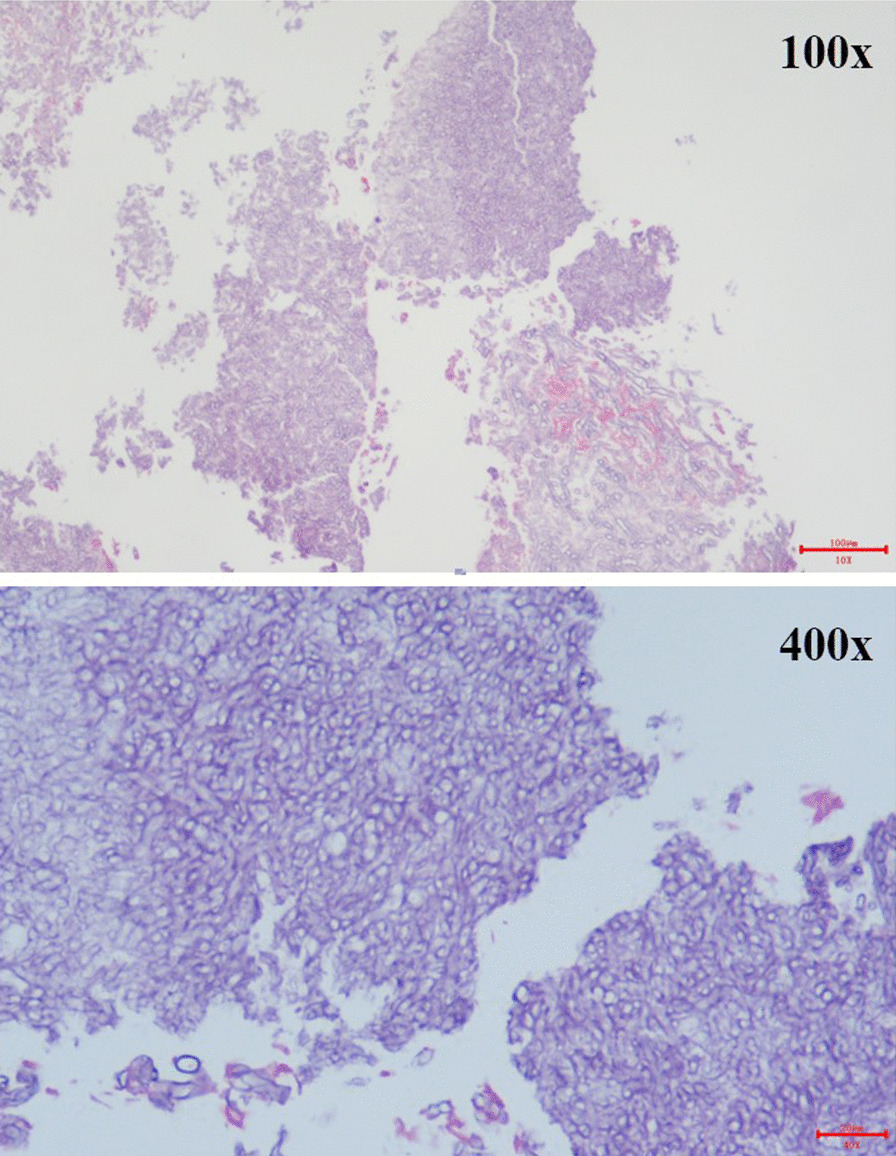
Detection of Aspergillus in lung biopsy sample of one patient in 100× and 400× magnitude respectively

Diagnosis results: visualized by images, majority of walls of cavities were thin. One patient had pleural wedge shape, and one developed pneumothorax. With the typical CT signs of IPA such as cavitied nodular and halo signs appearing on second CT scan, eight patients met the criteria of probable IPA, and two patients met the criteria of the possible IPA (Table 5). Only one patient had large peripheral nodules which allowed a biopsy through CT guidance. The lung tissue revealed *Aspergillus* (Fig. 2).

**Table 5:** Final diagnosis and outcomes of patients

Case no.	Host factors	Mycological findings		Initial CT scan	Initial IPA diagnosis	Second CT scan	Biopsy	Modified IPA diagnose	Outcomings after antifungal treatment
		Serum G/GM (pg/ml, AU/ml)	Positive culture for Aspergillus						
1	MP (253 mg)	117/0.48	Aspergillus	Nonspecific	Uncertain	Cavities	NA	Probable IPA	Recovery
2	MP (1048 mg)	NA/positive*	Negative	Nonspecific	Uncertain	Cavities	NA	Probable IPA	Improve but died due to discontinue of treatment
3	MP (1040 mg) Asthma Prostatic cancer	NA/0.08	Negative	Nonspecific	Uncertain	Cavities	NA	Possible IPA	Recovery
4	MP (1032 mg) COPD	3820/ positive	Negative	Nonspecific	Uncertain	Cavities	NA	Probable IPA	Recovery
5	MP(2360 mg) Asthma	NA/0.31	Negative	Nonspecific	Uncertain	Cavities	NA	Possible IPA	Recovery
6	Postoperative esophagus cancer	NA/NA	*Filamentous fungi*	Nonspecific	Uncertain	Cavities	NA	Probable IPA	Unknown
7	MP (1200 mg)	NA/0.07	*Aspergillus*	Nonspecific	Uncertain	Cavities	NA	Probable IPA	Recovery
8	None	269.40/ positive	*Aspergillus*	Nonspecific	Uncertain	Cavities	NA	Probable IPA	Recovery
9	None	NA/negative	NA	Nonspecific	Uncertain	Cavities	*Aspergillus*	Proven IPA	Recovery
10	MP(1200 mg) Asthma	27.55/positive	NA	Nonspecific	Uncertain	Cavities	NA	Probable IPA	Recovery

MP: Methylprednisolone. G test: (1,3)-β-D-glucan test, GM test: Galactomannan test,*Before G/GM detection, piperacillin tazobactam was used in this case. NA: not applicable.

Treatment process and results (Table 5): eight of the nine patients were treated with voriconazole for 15 days to 6 months. One patient was treated with voriconazole at first, but had no response. So eight days later the patient was treated with posazonazole instead and had a good response. Among the nine patients who received treatment, eight of them recovered well through evaluation of symptoms and CT scan signs and survived, the survival rate was 88.89%. One patient recovered after 2 weeks of voriconazole therapy, but voriconazole was discontinued because of economic cost of voriconazole. The patient died after discharge. Only one patient refused antifungal therapy and was lost during the following visit.

## Discussion

Data from a German study showed, during the period from 1979 to 1992, the incidence of invasive mycosis increased by about 8 times, and IPA, as the most harmful type and the most fatal type of pulmonary aspergillosis infection, has gradually been paid attention by clinical researchers [13]. Clinically, IPA is generally divided into neutropenia and non-neutropenia. This study mainly discusses the diagnostic methods of IPA patients with non-neutropenia. We found that IPA in nonneutropenic patients showed a specific progressive deterioration in the CT scan in a short interval, which promoted early diagnosis and timely antifungal therapy. The diagnosis of IPA was validated finally by therapeutic response and/or biopsy. Therefore, our findings provide a noninvasive, feasible and effective strategy for early diagnosis of IPA with URD. To the best of our knowledge, the current report is the first to emphasize the diagnostic value of dynamic monitor of CT scans in IPA with URD.

Research data shows that IPA is commonly diagnosed in neutropenic patients, but also could be diagnosed in nonneutropenic patients with URD [4, 14]. In our data, all patients had no neutropenia but less severe forms of immunocompromise in lungs. Most of the reasons for their admission were COPD, asthma and pneumonia, complicated with prostate cancer, hospital acquired pneumonia (HAP), and most of them had underlying diseases such as coronary heart disease, hypertension, and diabetes. Some patients had a history of malignant tumors. So we suggest to keep IPA in mind when managing the patients in department of respiratory medicine. The URD of those patients could further increase the complexity of *Aspergillus* diagnosis. First, pulmonary diseases commonly share same symptoms and signs with IPA, such as fever, dyspnea, chest tightness, wheezing and sputum production. And those non-specific symptoms and signs could mask *Aspergillus* infections. Second, corticosteroids and broad-spectrum antibiotics are used commonly even overused in these population, which could further increase risk for IPA [3, 4]. Third, biomarkers and specific CT signs of IPA are not sensitive in nonneutropenic patients. The specific CT signs like cavity or air crescent are less common in nonneutropenic patients than in neutropenic patients [7]. In agreement with another report [15], the most common CT finding was consolidation. Such CT sign is non-specific and might correspond to a wide range of morbidities such as bacterial pulmonary infection, cardiac failure, aspiration pneumonia and so on. At last, patients’ poor clinical conditions like weakness, dyspnea, hypoxic respiratory failure and cardiac failure made invasive procedures such as lung tissue biopsy and bronchoscopy risky. As in our report, only one patient received biopsy. Yet tissue biopsy and lower respiratory tract samples for culture or GM tests in BALF are very specific for IPA [9, 16].

The GM is a universal polysaccharide component in the cell wall of aspergillus, which is a polyantigen. The GM appears in circulation about 1 week earlier than clinical symptoms and imaging abnormalities. Continuous monitoring of patients' serum GM levels is helpful for early diagnosis of IPA and timely medication. And the detection of the GM antigen in BALF and serum serves as a reliable assay for the diagnosis of IPA [15]. Positive GM test has been taken as an important criterion for the diagnosis of IPA both by the EORTC/MSG and Bulpa criteria. In our report, only four out of nine patients reported positive GM test. So our results showed that GM assay has relatively low sensitivity in nonneutropenic patients, as reported previously [17, 18]. Meanwhile, there were other factors affecting result of GM test. One of the GM positive patients had been administered piperacillin-tazobactam prior to the test, which was reported to be one of the reasons for false positives in the serum-GM assay [19, 20]. Some studies have reported that the BALF-GM assay is more sensitive than the serum-GM assay and fungal cultures [17, 19, 21]. This is a shortcoming that bronchoalveolar lavage (BAL) was not conducted through bronchoscope as common in our study. A number of reasons prevented doctors to successfully obtain BALF. First, the bad general condition of patients, extreme discomfort and side effects of bronchoscope reduced patients’ compliance. Second proper standardization techniques of BAL are still lacking. There were variations in the BALF volumes and GM cut-off values reported in different studies. Moreover, the yield of BALF-GM is associated with the lavage site. Therefore, how to accurately locate the lesion is critical yet very difficult.

Previous studies reported that some special signs in CT are highly suggestive of IPA, like cavity, vessel occlusion signs [22], patchiness [23], airway-invasive features in nonneutropenic cases [24]. But several papers have reported that CT signs in nonneutropenic IPA is nonspecific. So the imaging findings of nonneutropenic IPA need further study.

First, it is reported that IPA in nonneutropenic patients have different tissue injury pathogenesis compared with neutropenic patients. Berenguer *et al* reported that nonneutropenic immunocompromised animals revealed a pattern of inflammatory necrosis but no significant angioinvasion, hemorrhage or infarction histologically demonstrated in persistently neutropenic animals with IPA [25]. The same tissue injury pattern was found in IPA patients [26]. It means the nonneutropenic patients should have the corresponding CT scans of tissue necrosis like cavities or halo signs.

Secondly, given IPA was an infectious disease, it might evolve over several phases which might begin with colonization, progress to infection and, finally lead to manifestation of disease symptoms in patients [27]. This phase evolving was reported in a female case of invasive tracheobronchial *Aspergillosis*, in which the CT scan was ordered on day 1, day 4, day 7, day 21, day 63 and day 139. It was found that invasive tracheobronchial *Aspergillosis* could progress to IPA with extended parenchymal lesions within a short period [28]. In summary, the CT signs in nonneutropenic IPA might change over time, and specific signs could appear in one certain time point. As showed in our report, IPA underwent a progress beginning with nonspecific CT signs, then developing to cavities within a short period of about 9 days, which was reported as appearing 2 weeks in neutropenic IPA [29]. Until now, this is the first report about the progress deterioration of CT scans in nonneutropenic IPA, the exact dynamic changes of CT scans in nonneutropenic IPA are far from clear, so specific study designed to observe CT signs at different stages of IPA is warranted.

Right now there are several guidelines of diagnosis and treatment for IPA released by several committees, namely EORTC/ MSG criteria [9], Bulpa criteria [30] and ICU criteria [30]. The scope for each guideline are different. EORTC/ MSG criteria is limited for cancer patients but also widely used in other patients. The Bulpa criteria is proposed to diagnose IPA specifically in COPD patients. The ICU criteria is proposed to diagnose IPA in the ICU setting. Items required for proven IPA are the same in the three sets of criteria, yet the items required for probable IPA are different. Here we used EORTC/ MSG criteria to diagnose IPA. When patients had a history of severe COPD, Bulpa criteria were also used. As we found, EORTC/ MSG criteria has strict requirements regarding the typical CT findings. So according to EORTC/ MSG criteria, probable/putative IPA should meet one of three CT signs in clinical data as follows, (a) Dense, well-circumscribed lesion(s) with or without a halo sign. (b) An air-crescent sign. (c) A cavity. Yet those typical CT signs for IPA (e.g. halo or air-crescent sign) are particularly rare in early stages in nonneutropenic patients. As showed in our study, the first CT scan only had some nonspecific CT signs as reported before [2, 8, 31], which were not helpful for early diagnosis and timely treatment.

Meanwhile, we found there was no requirement for dynamic changes of clinical exacerbation, neither the CT scan nor mycological findings in the EORTC/ MSG criteria. We speculated that it was because of EORTC/ MSG criteria mainly serving for cancer or hematopoietic malignancies, which might deteriorate in hours and days. Yet in IPA in nonneutropenic patients with local airway impaired immunity, the clinical process is not usually so urgent. Nousheen and colleagues reported the average length of hospital stay were 10.61 ± 9.08 days [32], and ours were 45.3 days. We found there was a very significant CT sign deterioration among those patients after average intervals of 9 days, at least 5 days. Our results suggested that EORTC/ MSG criteria were not sensitive enough for nonneutropenic IPA without reexamination of CT scans. Thus, the procedure of applying dynamic monitor of clinical or dynamic CT scans is a way to optimize the EORTC/ MSG criteria.

However, our study had several limitations. The diagnosis of invasive pulmonary aspergillosis should be confirmed by pathology, but in this study only one patient had pathology to confirm the diagnosis. And we only included 10 patients in the study, so the sample size was small. Further in-depth studies with large samples were needed to verify the validity of CT scan within short interval for IPA diagnosis.

## Conclusions

IPA in nonneutropenic patients with URD has become a challenge in clinical practice. By dynamically monitoring disease progression via CT, it might improve the accuracy of diagnosis, especially in seriously-ill patients who could not stand bronchoscopy and lacking positive mycological findings. We suggest the interval of CT scans could be around a week, or at least 5 days in emergency situation based on our data. Our novel finding might provide a valuable noninvasive and efficacious strategy in nonneutropenic IPA.

## Data Availability

The datasets generated and/or analysed during the current study are not publicly available due other manuscripts will be published from this data, but are available from the corresponding author on reasonable request.
